# A Diversity of Conserved and Novel Ovarian MicroRNAs in the Speckled Wood (*Pararge aegeria*)

**DOI:** 10.1371/journal.pone.0142243

**Published:** 2015-11-10

**Authors:** Shan Quah, Casper J. Breuker, Peter W. H. Holland

**Affiliations:** 1 Department of Zoology, University of Oxford, South Parks Road, Oxford, United Kingdom; 2 Evolutionary Developmental Biology Research Group, Department of Biological and Medical Sciences, Faculty of Health and Life Sciences, Oxford Brookes University, Gipsy Lane, Headington, Oxford, United Kingdom; Sars International Centre for Marine Molecular Biology, NORWAY

## Abstract

microRNAs (miRNAs) are important regulators of animal development and other processes, and impart robustness to living systems through post-transcriptional regulation of specific mRNA transcripts. It is postulated that newly emergent miRNAs are generally expressed at low levels and with spatiotemporally restricted expression domains, thus minimising effects of spurious targeting on animal transcriptomes. Here we present ovarian miRNA transcriptome data for two geographically distinct populations of the Speckled Wood butterfly (*Pararge aegeria*). A total of 74 miRNAs were identified, including 11 newly discovered and evolutionarily-young miRNAs, bringing the total of miRNA genes known from *P*. *aegeria* up to 150. We find a positive correlation between miRNA age and expression level. A common set of 55 miRNAs are expressed in both populations. From this set, we identify seven that are consistently either ovary-specific or highly upregulated in ovaries relative to other tissues. This ‘ovary set’ includes miRNAs with known contributions to ovarian function in other insect species with similar ovaries and mode of oogenesis, including miR-989 and miR-2763, plus new candidates for ovarian function. We also note that conserved miRNAs are overrepresented in the ovary relative to the whole body.

## Introduction

MicroRNAs (miRNAs) are short regulatory RNAs with important roles in post-transcriptional regulation [1]. They are of significance at all stages during an animal’s life cycle from embryonic development [2–5] to post-embryonic development [6,7] and reproduction [5,8]. Understanding the evolution of miRNAs is important to understanding the evolution of growth, reproduction and development.

It has been postulated that newly emergent animal miRNAs are generally expressed at low levels and with spatiotemporally restricted expression domains [9,10]. For example, in a study of miRNA expression in human and chimpanzee brains, more recently acquired miRNAs show a tendency for weaker expression than older miRNAs [10,11]. In mammals, a comprehensive study across multiple tissues demonstrates a positive correlation between miRNA age and expression levels [12].

The Speckled Wood butterfly (*Pararge aegeria*) is an ideal insect species with which we can address similar questions concerning miRNA evolution due to the availability of genome [13], transcriptome [14] and miRNA sequencing [15] data. Here we focus on miRNAs expressed during oogenesis, which are likely to include regulators of egg production and ovary function. Oogenesis in insects must generate a viable egg capable of initiating and sustaining embryonic development after fertilisation [16]. In addition to sustaining the energetic requirements for embryogenesis (17), the egg must also be pre-patterned with a polarity, which in insects occurs largely in the form of localised maternal determinants that drive zygotic spatiotemporal gene expression patterns essential for establishment of body axes [17]. Much is known about the identity and function of maternally provisioned mRNA molecules in insect eggs, but far less about other factors that influence the activity of such mRNAs, such as miRNAs. Furthermore, most of these studies have been performed in drosophilids [18], mosquitoes [19], cockroaches [20] and beetles [21]. Far less is known about the molecular control of oogenesis in butterflies and moths, despite Lepidoptera being one of the largest insect orders. Characterisation of the butterfly miRNA transcriptome would complement the current understanding of both genetic control involved in ovarian function [14] as well as the conservation of such mechanisms across insects.

## Materials and Methods

### Sample collection and sequencing

We sequenced the ovarian small RNA and mRNA transcriptomes of two females from each of two *P*. *aegeria* populations kept as lab stocks: (1) Zonza on Corsica, France, and (2) St Hubert, from the south of Belgium. The St Hubert population is the same population for which phenotypic data has previously been collected on maternal effects and egg production [22] as well as ovarian transcriptomic data [14] and for which a draft genome has been characterised [13]. The Zonza stock was newly established in 2014; the St Hubert stock has been in the lab since 2007 (see Carter et al. [14] for details regarding rearing *P*. *aegeria* in general and for St Hubert specifically).

Fully eclosed females were allowed to mate, and were placed individually in netted cages (0.5 m^3^) along with a potted *Poa trivialis* grass plant for oviposition and an artificial flower containing a 10% honey solution. Females (kept at 21±0.3°C, LD 16:8) were sacrificed 6 days after mating, when they reached the peak of egg production [22]. Ovaries were dissected and cleared of fat and surrounding tissue (cf. Carter et al. [14]). Total RNA was extracted using TRIzol (Life Technologies). Total RNA samples and analysed by Illumina HiSeq 2000 sequencing at the Beijing Genomics Institute. Each sample was used to generate both a mRNA transcriptome library as well as a small RNA library. The mRNA transcriptome sequencing used paired-end reads of 2 x 100 nucleotides from a 160 bp insert library.

### miRNA identification

Small RNA data processing was carried out as previously described [15], except that instead of annotating known miRNAs by comparison to miRBase, precursors were initially scanned by BLASTn against the set of previously reported *P*. *aegeria* miRNAs [15]. The dataset used in the previous study [15] was generated by pooling RNA in equal amounts from various life-cycle stages of the Speckled Wood butterfly, thereby eliminating potential bias from any one tissue. Sequences without hits against this dataset were subject to additional processing to remove hits against repetitive elements and non-coding RNAs, as well as precursors with reads mapping only to one arm and those with <95% of reads on either arm with the same 5’ starting position. Only sequences with >50 dominant arm reads in Zonza or >18 dominant arms in St Hubert were retained for annotation. The introduction of a minimal read count is because in this study we are focussing on miRNAs with putative ovarian function, whereas in Quah et al. [15] we were searching for all miRNAs; the minimal read counts chosen for the two samples are equivalent after compensating for relative miRNA sequencing depth. All shortlisted candidates were then compared against miRBase 20 to identify known miRNAs which were not have been found in the original *P*. *aegeria* miRNA survey [15]. The same cutoff values described above were used to determine if a known miRNA was expressed in both ovarian samples.

Read counts for each mature product, as generated by miRDeep2 [23], were cross-checked by running the quantifier.pl script in the miRDeep2 package, using known *P*. *aegeria* miRNA precursors for read mapping. Both approaches generated similar read count data. Raw read counts for the Zonza and St Hubert samples, as well as for the original pooled dataset, were normalised against the total number of miRNA reads in each sample.

### Transcriptome assembly

To assemble a reference ovarian mRNA transcriptome, raw RNAseq reads were processed at source for removal of low quality reads and adaptor trimming. Read quality was then validated by checking FASTQ data using FASTQC (http://www.bioinformatics.babraham.ac.uk/projects/fastqc) and the first 16 bases trimmed off all reads. Reads from Zonza and St Hubert were pooled and assembled using the Trinity assembler (version: trinityrnaseq_r20140717) [24] to generate a combined *P*. *aegeria* ovarian transcriptome assembly containing 54,861 contigs. Contig FPKM (Fragments Per Kilobase of exon per Million of fragments mapped) values for each sample were determined using RSEM [25]. In order to retain only biologically interpretable contigs, we employed a multi-step approach to annotation. Contigs were first screened by BLASTn against the nr database on NCBI to reduce the number of misassembled contigs in the dataset. Remaining contigs were then compared by BLASTn against the *P*. *aegeria* ovarian transcriptome reported by Carter et al. [14]. A referenced list of proteins with known roles in ovarian and egg function [14] was also used to screen the combined transcriptome assembly by tBLASTn. All BLAST searches were subject to an E-value cutoff of 1e-05. Only contigs with hits to one or more of these datasets were retained, leaving 17,636 annotated contigs (S1 Table) which were further characterised using Blast2GO [26]. This involved screening contigs by BLASTx followed by mapping and annotation. InterProScan and ANNEX were used to augment annotation of GO terms. GO terms were condensed using the generic GO Slim subset.

Raw data for both miRNA and total RNA sequencing are available via the Gene Expression Omnibus (accession: GSE72090) along with the combined ovarian transcriptome assembly.

### Statistical analyses

Total read counts (S2 Table) for miRNAs in each of the datasets were used for statistical analysis. miRNAs were designated ‘lineage-specific’ if found only in *P*. *aegeria* and ‘conserved’ if present in any other species. A two-tailed Mann-Whitney U test was conducted to determine if expression levels differed significantly between conserved and lineage-specific miRNAs in any of the datasets. We also conducted a Spearman’s rho analysis to examine correlation between miRNA age and expression level.

### miRNA target prediction

miRNA target prediction was carried out using the PITA algorithm [27] with default parameters. Only predictions with a score of -10 or below were accepted as predicted hits.

## Results

### Small RNA sequencing and data processing

We constructed and sequenced small RNA libraries from ovaries of the Speckled Wood butterfly *P*. *aegeria* to identify miRNAs expressed in the lepidopteran ovary. To allow for possible differences dependent on geography, climate or diet, we analysed females from two distinct populations from different parts of Europe: St Hubert in Belgium and Zonza in Corsica. Total RNA was extracted from ovaries and used to construct small RNA libraries for each sample. A total of 46,481,949 Illumina HiSeq reads were obtained (22,604, 869 for Corsica and 23,877,080 for Belgium). After filtering reads to remove possible degradation products and non-miRNAs, these were compared to a published dataset of *P*. *aegeria* miRNAs [15].

**Fig 1:**
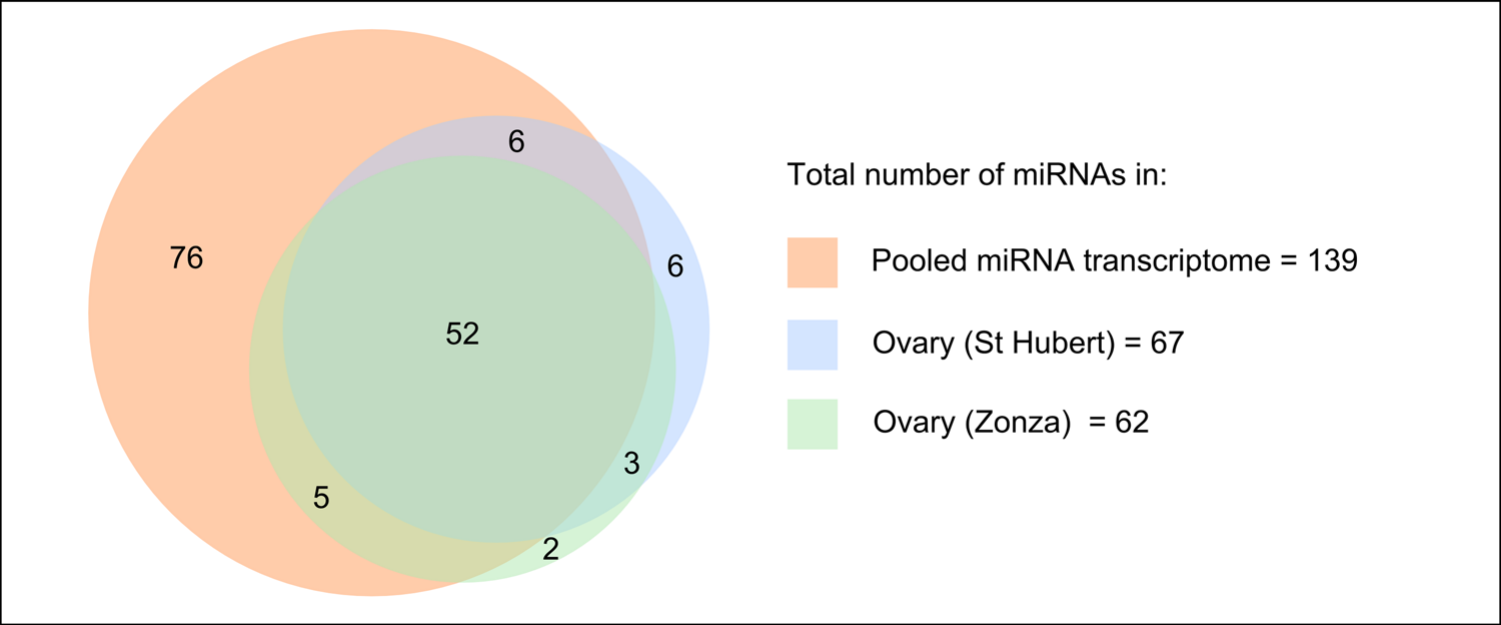
Distribution of miRNAs in each small RNA library.

Of the 139 previously known miRNAs in *P*. *aegeria* [15] we obtained reads for 80 and 76 miRNAs in the ovary-specific samples for St Hubert and Zonza respectively (S2 Table). Some of these were expressed at low levels and should not be considered as candidates for ovarian function; using read-count cut-off thresholds, we reduced this number to 58 and 57 in St Hubert and Zonza respectively. Of these, 52 known miRNAs are expressed above cutoff threshold in both populations (Fig 1, S2 Table). In addition, a total of 11 miRNAs that had not been previously described in *P*. *aegeria* were found; 2 were present in the Zonza sample only, 6 in the St Hubert sample only, and 3 in both (B-0070/C-0072, B-0210/C-0160 and B-0401/C-0173; Table 1). The total number of miRNAs identified as expressed in *P*. *aegeria* ovary is therefore 74 (63 previously described miRNAs and 11 newly discovered), with a total of 55 miRNAs expressed in the ovary in both populations.

**Table 1:** miRNAs identified in this study which were not found in a whole body pooled transcriptome reported earlier [15].

ID	Primary sequence	5' mature product	3' mature product
B-0401/C-0173	agcugugcuauauuacaauccucaacauuuuccaaaguagugggcguaauuuaucgugagcuagcuauagauauuaugcucauuacuuuggauauguuguggaacuuacauc	uccaaaguagugggcguaauuuau	auauuaugcucauuacuuuggau
B-0042	guuuuuucuacacaagauguuugucagugacaguugucacucacucaguagacacguuuauauuguccugucacuggguguaguguuuacuaucacuguaacggacacuccau	guugucacucacucaguagaca	ucacuggguguaguguuuacuau
B-0070/C-0072	ugcugcaaugaaaagaagaacaaucuccauugguaguugucacucacucaguagacuagucauaaaccagucacugggugucugauuacuaucacuggugaucauuaccau	guugucacucacucaguagacu	ucacugggugucugauuacua
B-0112	acaaggucuuuaucguugacaguugucacucacucagaagacacguuaauauuguucugucacuggguguagugauuacuauc	guugucacucacucagaagaca	ucacuggguguagugauuacuau
B-0164	gcaguuuuacuuugaggguguuguuuaauuuuggccaguagaaguucuucaaaacuuauugcuucauuugguuucguuaguucaggauuuucauccccgguggacauagauaagu	uguuuaauuuuggccaguagaagu	ucauuugguuucguuaguucagga
B-0210/C-0160	caagaaguuugucagugacaguugucacucgcucaguagacacguuaauaauguucugucacuggguguagugauuacuaucaauguaauauauacuccaucacaaaaaaaa	guugucacucgcucaguagaca	ucacuggguguagugauuacu
B-0218	agugacaguugucacacacucaguagacacguuuauauuguccugucacuggguuuagugauuacuaucacuguaacggacacuccau	guugucacacacucaguagaca	ucacuggguuuagugauuacuau
B-0298	uaaaaguaggaauuaugagagagagaauuacggacauuggcgucauuauaaucagaugaccggaccagaucaguauacucgcucuggauacuacacacauccuaaucaacagg	gagagaauuacggacauuggcgu	cagaucaguauacucgcucugga
B-0319	uagggauaggaaauaacauuuucucuuuagcaaaaguuaucauucacucaguaggcuugucaucaaucagucacuggguauaugauuacuaucgcuaaugaucacaucgac	aaguuaucauucacucaguaggcu	ucacuggguauaugauuacua
C-0149	aaggucuuuaucguugacaguugucacucacucagaagacacguuaauauuguucugucacuggguguagugauuacuaucaaugcaauauacacuccauaacuacgaaca	guugucacucacucagaagaca	ucacuggguguagugauuacu
7C-0261	ugucauuuggccccugaaauaauuggcacauguuauuuuuguagcugguuggcccauaugugcucaacuauggauuaugaagcaaagaaugcaagugucuuuuuaaaaugucaa	uauuuuuguagcugguuggcccau	uggauuaugaagcaaagaaugcaa

### Effect of miRNA age on expression level

We categorised the miRNAs present in the updated *P*. *aegeria* dataset by age, defining ‘conserved’ miRNAs as those present in *P*. *aegeria* and at least one other species and ‘lineage-specific’ miRNAs as those not found (or predicted in genome sequence) outside of the Speckled Wood. To test if the 11 newly discovered miRNAs are encoded by lineage-specific genes, or are evolutionarily older, each was investigated by using BLASTn against all known miRNA precursors on miRBase and their inferred pre-miRNA sequences were compared by BLASTn against the available genomes of other Lepidoptera (*Heliconius melpomene*, *Danaus plexippus*, *Manduca sexta*, *Bombyx mori*, *Plutella xylostella* and *Cameraria ohridella*) as well as that of the caddisfly (*Glyphotaelius pellucidus*). One sequence, B-0401/C-0173, was orthologous to miR-2763 (S1 Fig), which likely originated in the common lepidopteran ancestor [15], but was not previously identified in a whole body small RNA library for *P*. *aegeria* pooled across multiple developmental stages including adult females [15]; the other 10 sequences are thus far specific to *P*. *aegeria* and inferred to have arisen more recently. Combining these data with the 139 known *P*. *aegeria* miRNAs and their times of origin gives a total of 83 conserved and 67 lineage-specific miRNAs in this species.

**Fig 2:**
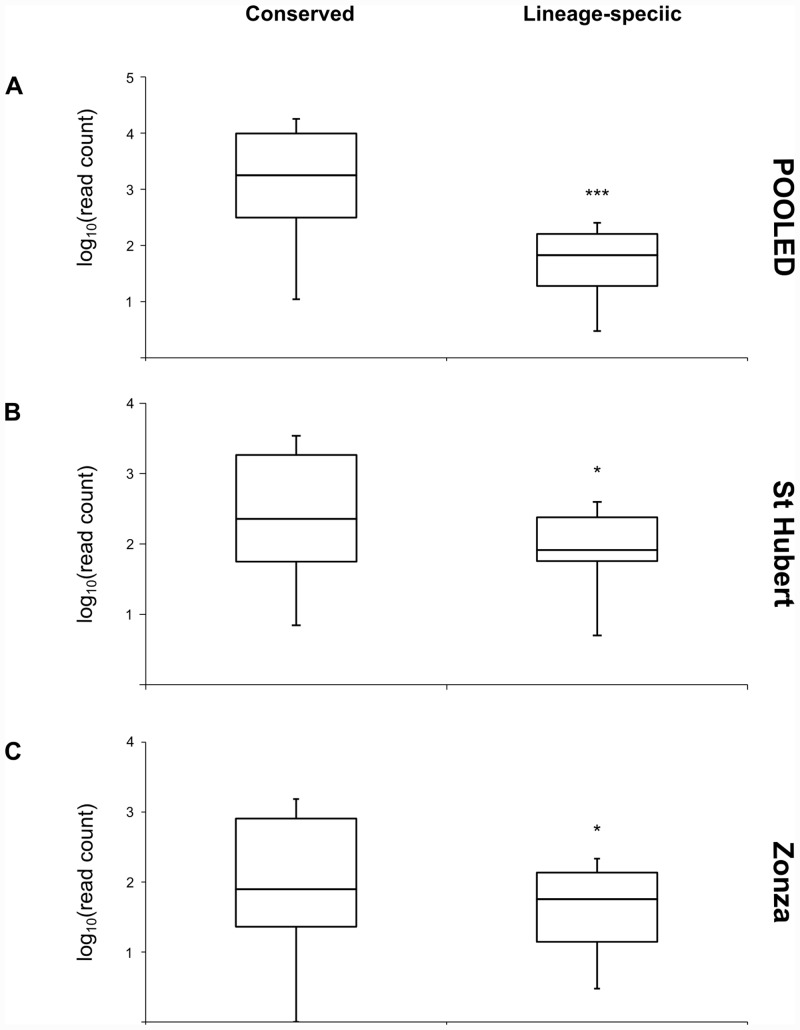
Comparison of miRNA expression levels. Distribution of expression levels for conserved and lineage-specific miRNAs in (A) pooled miRNA transcriptome, (B) St Hubert and (C) Zonza.

The total number of reads mapped to each miRNA precursor was taken as a measure of expression level and compared between conserved and lineage-specific miRNAs in St Hubert, Zonza, and the pooled miRNA transcriptome reported in Quah *et al* [15]. There is a large spread of expression levels detected for both conserved and lineage-specific miRNAs in each of the three datasets. A significant difference in median expression levels was observed between conserved and lineage-specific miRNAs (two-tailed Mann-Whitney U test; U(148) = 802.5 (*P* << 0.001; pooled sample), U(148) = 2134 (*P* < 0.05; St Hubert), U(148) = 1798 (*P* < 0.05; Zonza)) in each of the transcriptomes (Fig 2). In addition, the miRNAs with the highest expression levels in each dataset are all members of deeply conserved miRNA gene families, and there is a statistically significant correlation between miRNA age and expression level (Correlation analyses, Spearman’s rho = 0.582 (*P* << 0.001; pooled sample), 0.221 (*P* < 0.05; St Hubert), and 0.320 (*P* << 0.001; Zonza)).

### Older miRNAs are enriched in the ovary

**Fig 3:**
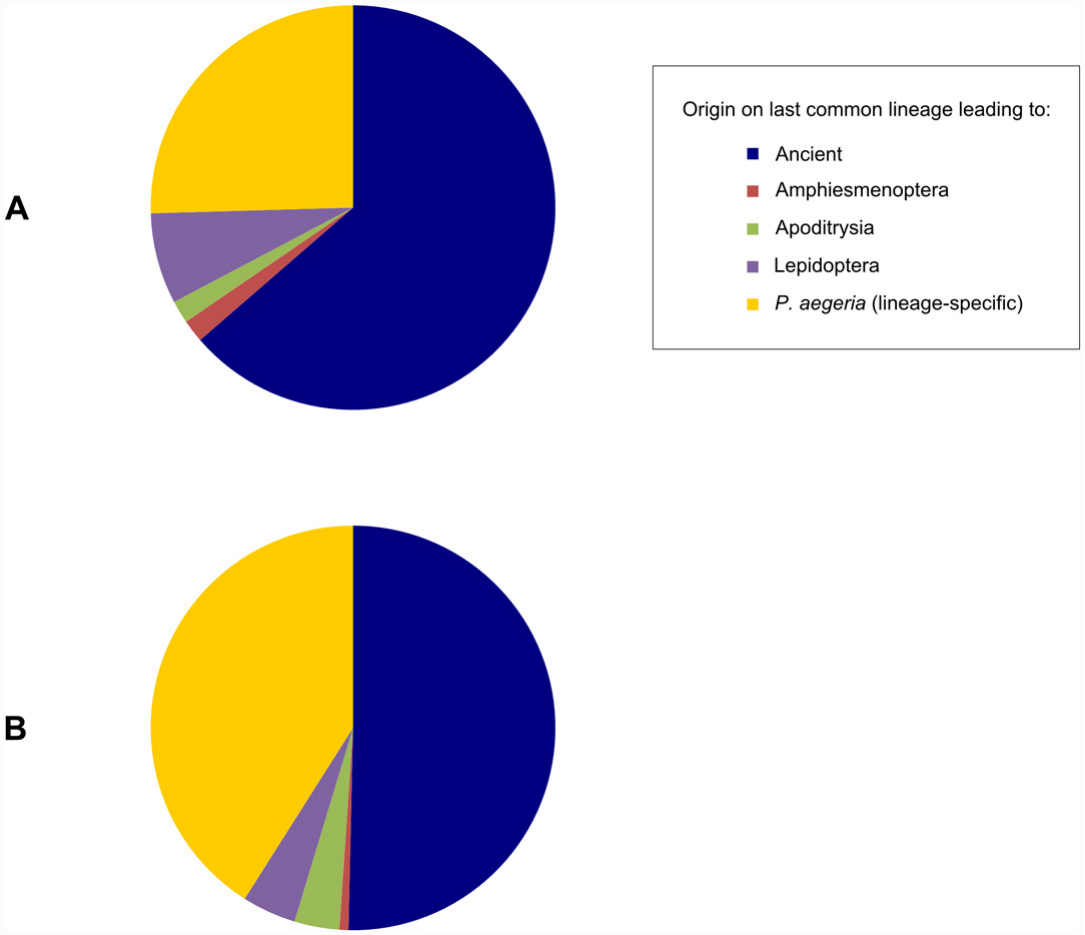
Proportion of miRNAs by age in *P*. *aegeria* whole body and ovarian transcriptomes. Pie chart showing the proportions of conserved miRNAs originating on each lineage which are (A) present in both St Hubert and Zonza ovarian transcriptomes and (B) present in the original pooled transcriptome reported in Chapter 3. ‘Ancient’ miRNAs are defined as those originating prior to the emergence of the Amphiesmenoptera (clade consisting of Lepidoptera and Trichoptera).

We studied relative proportions of conserved and lineage-specific miRNAs expressed in the ovary and compared these to the proportions observed in the pooled transcriptome (Fig 3). Ovarian expression of each miRNA was defined by its detection above cut-off levels in both populations. There is a higher proportion of conserved miRNAs (64%) expressed in the ovary compared to that in the pooled miRNA transcriptome (47%, chi-square *P* < 0.05).

### Ovarian-specific and ovarian-upregulated miRNAs

The most abundant miRNAs identified in the ovary are (in decreasing order of abundance) miR-989, miR-263a and miR-1. This observation is consistent between St Hubert and Zonza. Other consistently highly-expressed ovarian miRNAs include miR-993 and miR-184 (Table 2). In the pooled whole-body miRNA transcriptome, miR-1 is the most abundantly expressed sequence, followed by miR-989 and let-7; miR-263a and miR-993 are relatively highly expressed in the pooled transcriptome.

**Table 2:** The ten most abundant miRNAs ranked by expression in the whole body pooled transcriptome and each of the St Hubert and Zonza ovarian transcriptomes.

Rank	Whole body (pooled)	Ovary (St Hubert)	Ovary (Zonza)
1	Pae-miR-1	Pae-miR-989	Pae-miR-989
2	Pae-miR-989	Pae-miR-263a	Pae-miR-263a
3	Pae-let-7	Pae-miR-1	Pae-miR-1
4	Pae-miR-184	Pae-miR-184	Pae-miR-2755
5	Pae-miR-2766	Pae-miR-993	Pae-miR-184
6	Pae-miR-10a	Pae-miR-2755	Pae-miR-993
7	Pae-miR-31	Pae-miR-279d	Pae-let-7
8	Pae-miR-2755	Pae-miR-279b	Par-017
9	Pae-miR-263a	Par-017	Par-013
10	Pae-miR-281	Pae-let-7	Pae-miR-279b

**Fig 4:**
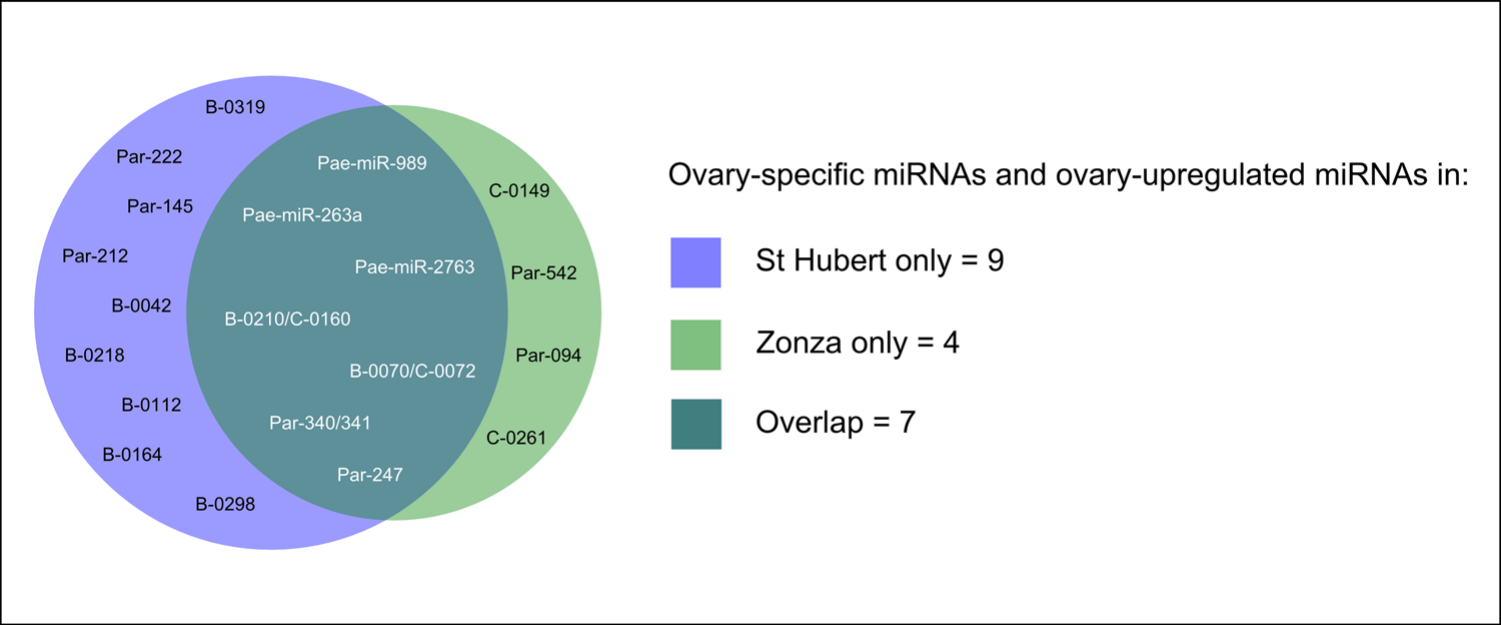
miRNAs with higher abundances in the ovarian datasets relative to the pooled miRNA transcriptome.

The above analyses reveal which miRNA genes are highly expressed in ovary, but this list includes miRNAs expressed in a range of tissues, as well as ovarian-upregulated sequences. To gain insight into the role of miRNAs in ovarian function, we wished to identify the latter category of miRNAs. Mature and dominant arm read counts for all miRNAs were normalised against the total number of miRNA reads per sample, and then compared with normalised read counts from the pooled miRNA dataset for *P*. *aegeria* from Quah et al. [15]. miRNAs with dominant arm read counts below a cutoff value in the Belgian and Corsican samples were deemed to not be expressed and excluded from analysis. These analyses identified five miRNAs with > 4-fold upregulation in both libraries (Table 3): Pae-miR-989, Pae-miR-263a, Par-247, Par-340 and Par-341. An additional 13 miRNAs showed up-regulation in just one ovary sample, or were only detected in either ovarian dataset but not in the pooled miRNA transcriptome (Fig 4, S2 Table). As Par-340 and Par-341 share 3’ arm reads, we group them together (Par-340/341) for the purpose of analysis. These four ‘ovarian-upregulated’ miRNA sequences can be combined with the three newly identified ‘ovary-specific’ miRNAs common to both samples (described above), to form a category of seven ‘ovary set miRNAs’ for further analysis.

**Table 3:** The ‘ovary set’ miRNAs which show greater than 4-fold abundance in both St Hubert (Belgium) and Zonza (Corsica) ovarian transcriptomes relative to the whole body transcriptome. Fold change is calculated from normalized total read. An accurate fold change in abundance cannot be given for the last three miRNAs on the table, as these were detected only in ovarian transcriptomes (both populations) and not at all in the pooled transcriptome.

	Ovary (St Hubert)				Ovary (Zonza)			
miRNA	Total	5'	3'	Fold change	Total	5'	3'	Fold change
Par-247	125	120	5	27.5	146	142	4	11.5
Par-341	52	51	1	10.5	70	69	1	5.0
Par-340	52	51	1	10.3	70	69	1	4.9
Pae-miR-989	288099	32	288067	5.2	873884	88	873796	5.6
Pae-miR-263a	24821	24819	2	4.5	63593	63591	2	4.1
B-0070/C-0072	291	173	118	-	574	130	444	-
B-0210/C-0160	97	9	88	-	262	19	243	-
B-0401/C-0173 (Pae-miR-2763)	27	6	21	-	137	8	129	-

### Identification of putative ovarian miRNA targets

To identify possible mRNAs which may be regulated by the ovary-set miRNAs, we ran target predictions for these miRNAs against ovarian transcriptomes constructed from the same *P*. *aegeria* total RNA extraction. The Illumina HiSeq 2000 platform was used to generate 26,332,892 read pairs for Zonza and 26,466,283 read pairs for St Hubert.

Reads from both samples were pooled to construct a combined transcriptome assembly (Table 4, S1 Table).The PITA target prediction algorithm [27] was used to identify potential targets in the transcriptome for both 5’ and 3’ mature products for each of the 7 ‘ovary-set’ miRNAs.

**Table 4:** Trinity assembly statistics for the separate St Hubert and Zonza mRNA transcriptomes as well as a transcriptome combining reads from both populations. Number of contigs is prior to filtering by BLAST to remove potentially misassembled contigs.

	Combined assembly	St Hubert	Zonza
Number of contigs	54861	40915	45206
Total length	64060799	47311956	51104606
Maximum contig length	16200	16174	14446
N50	2236	2295	2130
N90	439	423	426

**Fig 5:**
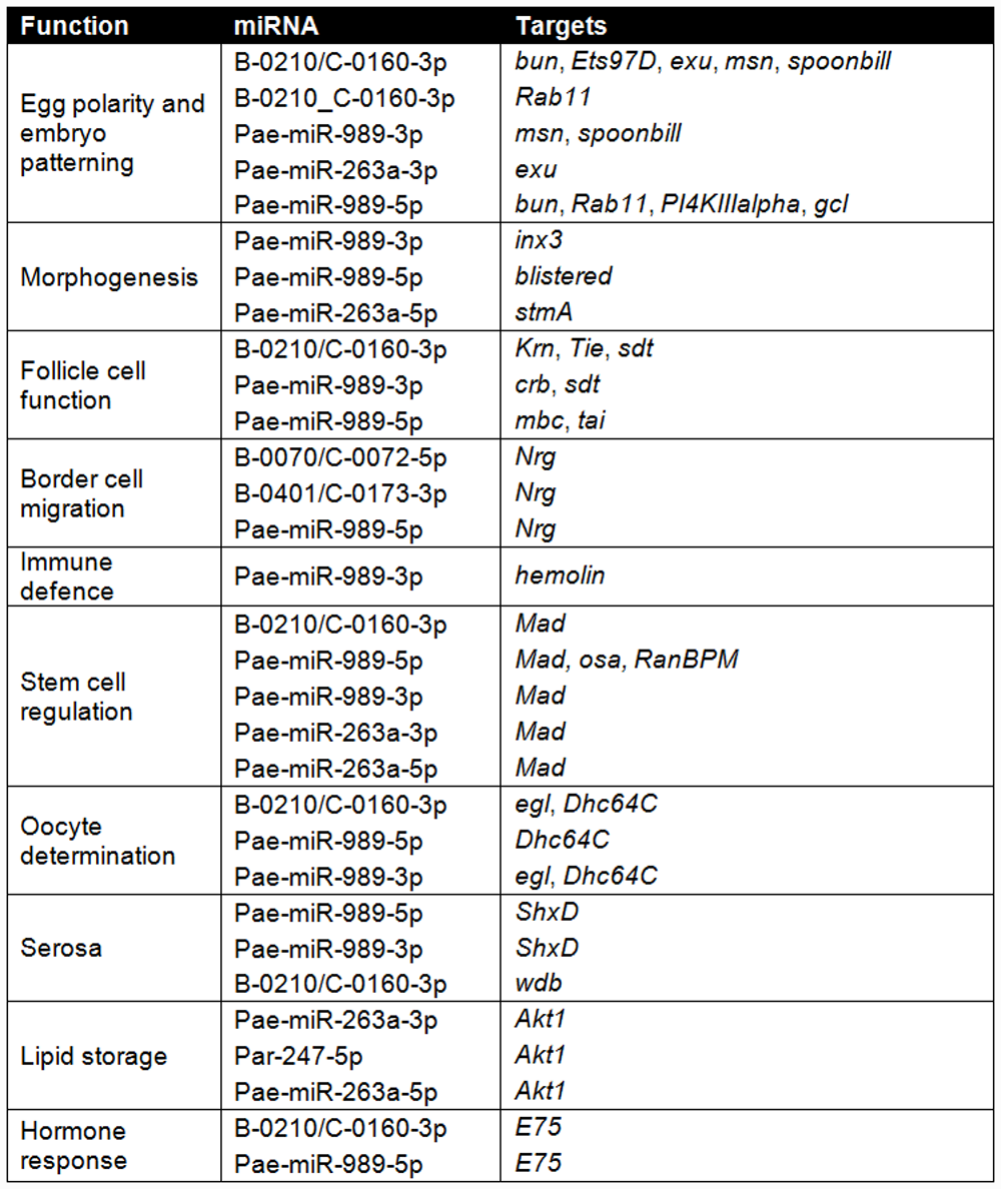
Predicted targets of ‘ovary set’ miRNAs with reported roles in ovarian and egg function.

We then sought to identify specific miRNA-mRNA target pairs of interest within the dataset. Predicted target contigs corresponding to genes involved in ovarian and egg function [14] are listed in Fig 5. These contigs cover a variety of functions including regulation of egg chamber development, oocyte provisioning and defence against pathogens and the external environment. In many cases, multiple ‘ovary set’ miRNAs are predicted to target the same transcript, an example of which is *neuroglian* (*nrg*) involved in regulation of border cell migration in flies [28].

## Discussion

### Diversity of miRNAs in *Pararge aegeria*

In a previous study, 139 miRNA genes were identified in the Speckled Wood butterfly, *P*. *aegeria* [15]. Through deep sequencing of small RNAs in adult ovaries from two geographically-distinct populations, we have now increased this number to 150 expressed miRNA genes. Of the 11 new miRNAs identified in the ovarian small RNA transcriptome, six are uniquely expressed in the St Hubert sample, two are unique to Zonza, and three were found in both populations.

A common set of 55 miRNAs are expressed in the ovaries of both St Hubert and Zonza. The most highly expressed of these include miRNAs also known to be highly expressed in other insects, such as miR-989 and miR-1. miR-1 is highly conserved across bilaterians [29] and has documented expression in muscle in vertebrates [30,31] and insects [32]. Its expression is also known to be highly abundant in the ovaries of the cockroach *Blatella germanica* [33]. miR-989 is known to be expressed in the ovary in mosquitoes [19] and flies [34].

Twenty-one of the 150 miRNA genes in the *P*. *aegeria* miRNA transcriptome are upregulated at least 4-fold in, or are specific to, the ovary in at least one sample. Seven of these are either upregulated or ovary-specific in both populations. We refer to these seven genes as the ‘ovary set’ miRNAs.

### miRNA age, ovary expression and expression level

New miRNAs tend to be expressed at low levels and in restricted spatiotemporal domains [9,10]. These characteristics would avoid severe fitness penalties due to the minimal requirements for miRNA-target binding and the resultant large number of potential targets in the transcriptome. Over time, deleterious target sites would be purged from the genome and advantageous target sites preserved, enabling the miRNA to increase its expression level and relax its tissue specificity.

Here we utilise our characterisation of the butterfly ovarian miRNA transcriptome, combined with our previous analysis of a pooled whole body and developmental miRNA transcriptome, to examine expression levels of conserved and lineage-specific miRNAs, as well as their relative contribution to the ovarian miRNA transcriptome. We consider separately the two ways in which overall miRNA expression might be increased—breadth of expression domains and level of transcription. Based on the model described above, we would predict that conserved miRNAs exhibit overall higher expression levels in the pooled transcriptome due to a wider range of expression domains. If evolutionarily-younger miRNAs were more tissue-specific miRNAs, these might be predicted to be under-represented in the pooled transcriptome, and reads might even be missed due to dilution effects.

We observed a larger proportion of conserved miRNAs expressed in the ovary compared to the pooled whole-body transcriptome, somewhat at odds with the predictions made by the model. One possible explanation for this is that the whole-body transcriptome includes RNAs from embryonic stages. It has been suggested that the early embryo represents a permissive environment for newly acquired miRNAs to be expressed and exert their effects [35]. Increased representation of young miRNAs in a library comprised of pooled developmental stages may result from such miRNAs being expressed during embryogenesis. An alternative explanation is that the capacity of a particular tissue to tolerate experimentation with novel miRNA expression may vary between tissues; tissues with highly complex transcriptomes such as the primate brain, which provides strong evidence for the above model [11], may differ in their robustness and resultant response to new miRNAs compared to those with simpler transcriptomes. Thus, it is possible that while some tissues express a large number of lineage-specific (young) miRNAs, new miRNAs arising under restricted transcriptional control in less permissive tissues are more likely to be eliminated by selection.

Conserved miRNAs are also predicted to display higher expression levels than lineage-specific miRNAs. A seminal study in mammals [12] reported a striking correlation between expression levels of miRNA families and age. In agreement with this report, deeply conserved miRNAs have the highest expression levels in all our datasets and a significant positive correlation exists between miRNA age and expression level. We also observe that both conserved and young miRNAs possess a wide range of expression levels, and that these ranges overlap substantially with each other. The observation that older lepidopteran miRNAs tend to exhibit higher expression levels is in line with the model of Chen & Rajewsky, and also replicates findings observed in mammals [12]. Our finding that some evolutionarily young miRNAs can also be expressed at high levels may also be reconciled with predictions of the model. Given the constant high turnover of young miRNAs in metazoan genomes [12,15,36], deep sequencing of a particular tissue presents a snapshot of the transcriptome at a given point in time. It is unlikely that there is any mechanistic bias behind miRNA birth to predispose newly-evolved miRNAs to emerge under the control of tissue-specific promoters with low expression levels, but it may be the case that such miRNAs are more likely to subsequently avoid elimination through natural selection.

### Potential conserved ovarian functions for insect miRNAs

A combined transcriptome assembly for both populations was constructed and used for miRNA target prediction on mature 5’ and 3’ products of all seven ‘ovary set’ miRNAs. One of the newly identified miRNAs within the ‘ovary set’ is the Speckled Wood homologue of miR-2763, previously described in the moths *B*. *mori* and *Manduca sexta* [37]. Three potential targets were identified for Bmo-miR-2763 in the *B*. *mori* genome [38]. The first encodes the diapause hormone receptor (*DHR*), expressed in the silkmoth ovary [39], where it is involved in transduction of the diapause hormone signal and exerts regulation over embryonic diapause. The second predicted target, *GRF*, encodes an insect protein related to vertebrate germ cell nuclear factor (GCNF). [40]. GRF may function in ecdysteroid signalling in moths [40]. The third putative target, *GATAβ*, is implicated in choriogenesis [38]. Although these target predictions were not replicated in *P*. *aegeria*, this may be due to partial mRNA predictions in the ovary transcriptome data, so some conservation of function is still possible.

Another miRNA with possible conserved ovarian functions across a wide variety of insect species is miR-989: a likely insect-specific miRNA [41] and the most abundant miRNA in the butterfly ovary. We found expression of this mRNA to be upregulated > 5-fold in both butterfly ovary samples relative to the pooled sample, and similarly it is highly expressed in ovaries of several fly species [42]; it is also upregulated in mosquito ovaries in response to blood feeding [19]. The pupal phase, during which oogenesis occurs, coincides with increased expression miR-989 in *B*. *mori* [38], suggestive of a similar upregulation of this miRNA during egg production in the Lepidoptera. In *Drosophila melanogaster* [34], it was demonstrated that a loss of miR-989 function impairs border cell migration in the egg chamber, leading to sterility.

miR-263a is the third ‘ovary set’ miRNA found in *P*. *aegeria* which has been characterised in a variety of other insect species. It has been hypothesised that miR-263a is involved in trans-generational immune priming in the greater wax moth *Galleria mellonella* [43]. It is strongly upregulated in *G*. *mellonella* pupae, and is also induced in the larval midgut, the rest of the body, and the eggs of females fed as larvae on diets contaminated with pathogenic bacteria. Our finding that miR-263a is highly expressed in the ovaries of *P*. *aegeria* lends support to the idea that a role in defence could be conserved across Lepidoptera, but more direct evidence is required to test this.

### Candidate targets and their involvement in butterfly ovarian biology

Further insights into the role of each miRNA on ovarian biology can be made by bioinformatic analysis of a curated list of genes implicated in egg and ovary function [14]. In each case, we suggest that the miRNA of interest may be as fine-tuners of gene activity rather than on/off switches for gene expression. This would grant robustness to the system and buffer it against developmental perturbation [44,45].

### Hormone signalling

The nuclear receptor E75 is an early response gene in the ecdysone signalling pathway and is involved in the ovarian response to hormone signalling in silkmoths [46] and flies [47]. We identified the *E75* mRNA splice variant *E75B* as a potential target for two of our ‘ovary set’ miRNAs (Fig 5). Both the predicted miR-989 and B-0210/C-0160 target sites are located at the start codon of this transcript.

### Follicle cell function

Follicle cells perform several key functions during insect oogenesis including egg provisioning [48], secretion of maternal ecdysteroids [49], and production of the chorion and eggshell. Follicle cells in flies also provide spatial patterning information to the oocyte [50].

In *Drosophila*, a subset of follicle cells (‘border cells’) contribute to both anterior patterning as well as the formation of the micropyle through which sperm enter the egg at fertilization [51]. Several ‘ovary set‘ miRNAs, including miR-989 as mentioned above, are predicted to target genes involved in follicle cell function and border cell migration. These include *stardust*, *crumbs* [52], *neuroglian* [28] and *taiman* (*tai*) [53].

### Serosa and immune defence

We predict that miR-989 may target the mRNA of *ShxD*, a homeobox gene expressed maternally in ovarioles and zygotically in the serosa [13]. The Shx genes are implicated in specifying the location of the extra-embryonic serosal tissue domain in developing oocytes, as well as regulating its subsequent development. As noted above, miR-263a may also be involved in immune defence, through different targets.

## Supporting Information

S1 Fig Alignment of miR-2763 sequences across Lepidoptera. (DOCX)

S1 Table Annotation of the combined mRNA transcriptome assembly. (XLSX)

S2 Table Read count comparisons for miRNA transcriptomes. (DOCX)

## References

[1] Behura SK (2007). Insect microRNAs: Structure, function and evolution. Insect Biochem Mol Biol 37(1):3–9. 10.1016/j.ibmb.2006.10.006 17175441

[2] Yekta S, Shih I-H, Bartel DP. MicroRNA-directed cleavage of HOXB8 mRNA (2004). Science 23;304(5670):594–6. 10.1126/science.1097434 15105502

[3] Pasquinelli AE, Hunter S, Bracht J. (2005). MicroRNAs: a developing story. Curr Opin Genet Dev. 15(2):200–5. 10.1016/j.gde.2005.01.002 15797203

[4] Jones CI, Newbury SF (2010). Functions of microRNAs in *Drosophila* development. Biochem Soc Trans. 38(4):1137–43. 10.1042/BST0381137 20659018

[5] Lasko P (2011). Posttranscriptional regulation in *Drosophila* oocytes and early embryos. Wiley Interdisciplinary Reviews: RNA. 2(3):408–16. 10.1002/wrna.70 21957026

[6] Kenny NJ, Quah S, Holland PWH, Tobe SS, Hui JHL (2013). How are comparative genomics and the study of microRNAs changing our views on arthropod endocrinology and adaptations to the environment? Gen Comp Endocrinol. 188:16–22. 10.1016/j.ygcen.2013.02.013 23480873

[7] Lozano J, Montañez R, Belles X (2015). miR-2 family regulates insect metamorphosis by controlling the juvenile hormone signaling pathway. Proc Nat Acad Sci. 112(12):3740–3745. 10.1073/pnas.1418522112 25775510 PMC4378413

[8] McBride D, Carré W, Sontakke SD, Hogg CO, Law A, Donadeu FX, et al (2012). Identification of miRNAs associated with the follicular-luteal transition in the ruminant ovary. Reproduction. 144(2):221–33. 10.1530/REP-12-0025 22653318

[9] Chen K, Rajewsky N (2007). The evolution of gene regulation by transcription factors and microRNAs. Nat Rev Genet. 8(2):93–103. 10.1038/nrg1990 17230196

[10] Berezikov E (2011). Evolution of microRNA diversity and regulation in animals. Nat Rev Genet. 12(12):846–60. 10.1038/nrg3079 22094948

[11] Berezikov E, Thuemmler F, van Laake LW, Kondova I, Bontrop R, Cuppen E, et al (2006). Diversity of microRNAs in human and chimpanzee brain. Nat Genet. 38(12):1375–7. 10.1038/ng1914 17072315

[12] Meunier J, Lemoine F, Soumillon M, Liechti A, Weier M, Guschanski K, et al (2013). Birth and expression evolution of mammalian microRNA genes. Genome Res. 23(1):34–45. 10.1101/gr.140269.112 23034410 PMC3530682

[13] Ferguson L, Marlétaz F, Carter J-M, Taylor WR, Gibbs M, Breuker CJ, et al (2014). Ancient expansion of the Hox cluster in Lepidoptera generated four homeobox genes implicated in extra-embryonic tissue formation. PLoS Genet. 10(10):e1004698 10.1371/journal.pgen.1004698 25340822 PMC4207634

[14] Carter J-M, Baker SC, Pink R, Carter DRF, Collins A, Tomlin J, et al (2013). Unscrambling butterfly oogenesis. BMC Genomics. 14:283 10.1186/1471-2164-14-283 23622113 PMC3654919

[15] Quah S, Hui JHL, Holland PWH (2015). A Burst of miRNA Innovation in the Early Evolution of Butterflies and Moths. Mol Biol Evol. 32(5):1161–1174. 10.1093/molbev/msv004 25576364 PMC4408404

[16] Bastock R, Johnston D St. *Drosophila* oogenesis. Curr Biol.2008 18(23):R1082–7. 10.1016/j.cub.2008.09.011 19081037

[17] Tadros W, Lipshitz HD. The maternal-to-zygotic transition: a play in two acts. Development. 2009;136(18):3033–42. 10.1242/dev.033183 19700615

[18] Barckmann B, Simonelig M. Control of maternal mRNA stability in germ cells and early embryos. Biochim Biophys Acta. 2013;1829(6–7):714–24. 10.1016/j.bbagrm.2012.12.011 23298642

[19] Mead EA, Tu Z. Cloning, characterization, and expression of microRNAs from the Asian malaria mosquito, *Anopheles stephensi* . BMC Genomics. 2008;9:244 10.1186/1471-2164-9-244 18500992 PMC2430712

[20] Chen W, Jiang GF, Sun SH, Lu Y, Ma F, Li B. Identification of differentially expressed genes in American cockroach ovaries and testes by suppression subtractive hybridization and the prediction of its miRNAs. Mol Genet Genomics. 2013;288(11):627–38. 10.1007/s00438-013-0777-1 23996145

[21] Preuss KM, Lopez J a, Colbourne JK, Wade MJ. Identification of maternally-loaded RNA transcripts in unfertilized eggs of *Tribolium castaneum* . BMC Genomics. 2012;13(1):671 10.1186/1471-2164-13-671 23181844 PMC3536564

[22] Gibbs M, Breuker CJ, Van Dyck H (2013). Flight during oviposition reduces maternal egg provisioning and influences offspring development in Pararge aegeria (L.). Physiol Entomol. 35(1):29–39. 10.1111/j.1365-3032.2009.00706.x

[23] Friedländer MR, Mackowiak SD, Li N, Chen W, Rajewsky N (2012). miRDeep2 accurately identifies known and hundreds of novel microRNA genes in seven animal clades. Nucleic Acids Res. 40(1):37–52. 10.1093/nar/gkr688 21911355 PMC3245920

[24] Grabherr MG, Haas BJ, Yassour M, Levin JZ, Thompson DA, Amit I, et al (2011). Full-length transcriptome assembly from RNA-Seq data without a reference genome. Nat Biotechnol. 29(7):644–52. 10.1038/nbt.1883 21572440 PMC3571712

[25] Li B, Dewey CN (2011). RSEM: accurate transcript quantification from RNA-Seq data with or without a reference genome. BMC Bioinformatics. 12:323 10.1186/1471-2105-12-323 21816040 PMC3163565

[26] Conesa A, Götz S, García-Gómez JM, Terol J, Talón M, Robles M (2005). Blast2GO: A universal tool for annotation, visualization and analysis in functional genomics research. Bioinformatics. 21(18):3674–6. 10.1093/bioinformatics/bti610 16081474

[27] Kertesz M, Iovino N, Unnerstall U, Gaul U, Segal E (2007). The role of site accessibility in microRNA target recognition. Nat Genet. 39(10):1278–84. 10.1038/ng2135 17893677

[28] Wei J, Hortsch M, Goode S (2004). Neuroglian stabilizes epithelial structure during *Drosophila* oogenesis. Dev Dyn. 230(4):800–8. 10.1002/dvdy.20108 15254915

[29] Wheeler BM, Heimberg AM, Moy VN, Sperling E a, Holstein TW, Heber S, et al (2009). The deep evolution of metazoan microRNAs. Evol Dev. 11(1):50–68. 10.1111/j.1525-142X.2008.00302.x 19196333

[30] Chen J-F, Mandel EM, Thomson JM, Wu Q, Callis TE, Hammond SM, et al (2006). The role of microRNA-1 and microRNA-133 in skeletal muscle proliferation and differentiation. Nat Genet. 38(2):228–33. 10.1038/ng1725 16380711 PMC2538576

[31] Kloosterman WP, Wienholds E, de Bruijn E, Kauppinen S, Plasterk RHA (2006). In situ detection of miRNAs in animal embryos using LNA-modified oligonucleotide probes. Nat Methods. 3(1):27–9. 10.1038/nmeth843 16369549

[32] Sokol NS (2005). Mesodermally expressed *Drosophila* microRNA-1 is regulated by Twist and is required in muscles during larval growth. Genes Dev. 19(19):2343–54. 10.1101/gad.1356105 16166373 PMC1240043

[33] Cristino AS, Tanaka ED, Rubio M, Piulachs M-D, Belles X (2011). Deep Sequencing of Organ- and Stage-Specific microRNAs in the Evolutionarily Basal Insect Blattella germanica (L.) (Dictyoptera, Blattellidae). PLoS One. 28;6(4):e19350 10.1371/journal.pone.0019350 21552535 PMC3084283

[34] Kugler JM, Chen YW, Weng R, Cohen SM (2013). miR-989 Is required for border cell migration in the *Drosophila* Ovary. PLoS One. 8(7): e67075 10.1371/journal.pone.0067075 23843983 PMC3700948

[35] Ninova M, Ronshaugen M, Griffiths-Jones S (2014). Fast-evolving microRNAs are highly expressed in the early embryo of *Drosophila virilis* . RNA. 20:360–372. 10.1261/rna.041657.113 24448446 PMC3923130

[36] Lyu Y, Shen Y, Li H, Chen Y, Guo L, Zhao Y, et al (2014). New microRNAs in Drosophila—birth, death and cycles of adaptive evolution. PLoS Genet. 10(1):e1004096 10.1371/journal.pgen.1004096 24465220 PMC3900394

[37] Zhang X, Zheng Y, Jagadeeswaran G, Ren R, Sunkar R, Jiang H (2012). Identification and developmental profiling of conserved and novel microRNAs in *Manduca sexta* . Insect Biochem Mol Biol. Elsevier Ltd; 42(6):381–95. 10.1016/j.ibmb.2012.01.006 PMC3340478 22406339

[38] Jagadeeswaran G, Zheng Y, Sumathipala N, Jiang H, Arrese EL, Soulages JL, et al (2010). Deep sequencing of small RNA libraries reveals dynamic regulation of conserved and novel microRNAs and microRNA-stars during silkworm development. BMC Genomics. 11:52 10.1186/1471-2164-11-52 20089182 PMC2824724

[39] Homma T, Watanabe K, Tsurumaru S, Kataoka H, Imai K, Kamba M, et al (2006). G protein-coupled receptor for diapause hormone, an inducer of Bombyx embryonic diapause. Biochem Biophys Res Commun. 344(1):386–93. 10.1016/j.bbrc.2006.03.085 16600181

[40] Charles JP, Shinoda T, Chinzei Y (1999). Characterization and DNA-binding properties of GRF, a novel monomeric binding orphan receptor related to GCNF and FTZ-F1. Eur J Biochem. 266(1):181–90. 10.1046/j.1432-1327.1999.00842.x 10542063

[41] Kozomara A, Griffiths-Jones S. miRBase: integrating microRNA annotation and deep-sequencing data. Nucleic Acids Res, 39:D152–7. 10.1093/nar/gkq1027 21037258 PMC3013655

[42] Lucas KJ, Myles KM, Raikhel AS (2013). Small RNAs: A new frontier in mosquito biology. Trends Parasitol. 29(6):295–303. 10.1016/j.pt.2013.04.003 23680188 PMC5739026

[43] Mukherjee K, Vilcinskas A (2014). Development and immunity-related microRNAs of the lepidopteran model host *Galleria mellonella* . BMC Genomics. 15(1):705 10.1186/1471-2164-15-705 25149864 PMC4156658

[44] Stark A, Brennecke J, Bushati N, Russell RB, Cohen SM (2005). Animal MicroRNAs confer robustness to gene expression and have a significant impact on 3’UTR evolution. Cell. 123(6):1133–46. 10.1016/j.cell.2005.11.023 16337999

[45] Ebert MS, Sharp PA (2012). Roles for MicroRNAs in conferring robustness to biological processes. Cell. 149(3):505–24. 10.1016/j.cell.2012.04.005 PMC3351105 22541426

[46] Papantonis A, Swevers L, Iatrou K (2015). Chorion Genes: A landscape of their Evolution, Structure, and regulation. Annu Rev Entomol. 10.1146/annurev-ento-010814-020810 25341099

[47] Ables ET, Bois KE, Garcia CA, Drummond-Barbosa D (2015). Ecdysone response gene E78 controls ovarian germline stem cell niche formation and follicle survival in *Drosophila* . Dev Biol. 400(1):33–42. 10.1016/j.ydbio.2015.01.013 25624267 PMC4448935

[48] Swevers L, Iatrou K (2003). The ecdysone regulatory cascade and ovarian development in lepidopteran insects: Insights from the silkmoth paradigm. Insect Biochem Molec. 33(121):1285–97. 10.1016/j.ibmb.2003.06.012 14599500

[49] Kadono-okuda K, Amornsak W, Yamashita O (1994). Controlled Ecdysteroid Accumulation in Eggs of the Silkworm, Bombyx mori, by an lmidazole Compound (KK-42), and Embryogenesis in These Eggs. Arch Insect Biochem. 135(1 994):121–35. 10.1002/arch.940250205

[50] Saunders C, Cohen RS (1999). The role of oocyte transcription, the 5’UTR, and translation repression and derepression in *Drosophila* gurken mRNA and protein localization. Mol Cell. 3(1):43–54. 10.1016/S1097-2765(00)80173-2 10024878

[51] Montell DJ (2003). Border-cell migration: the race is on. Nat Rev Mol Cell Biol. 4(1):13–24. 10.1038/nrm1006 12511865

[52] Pinheiro EM, Montell DJ (2004). Requirement for Par-6 and Bazooka in *Drosophila* border cell migration. Development. 131(21):5243–51. 10.1242/dev.01412 15456726

[53] McDonald JA, Pinheiro EM, Montell DJ (2003). PVF1, a PDGF/VEGF homolog, is sufficient to guide border cells and interacts genetically with Taiman. Development. 130(15):3469–78. 10.1242/dev.00574 12810594

