# Supplementary material for: A Diversity of Conserved and Novel Ovarian MicroRNAs in the Speckled Wood (Pararge aegeria)
Source: PLoS One. 2015 Nov 10;10(11):e0142243. doi: 10.1371/journal.pone.0142243 (PMC4640560; doi:10.1371/journal.pone.0142243)
Supplement: S1 Fig — (DOCX) [file pone.0142243.s001.docx]

B-0401/C-0173 AGCUGUGCUAUAUUACAAUCCUCAACAUUUUCCAAAGUAGUGGGCGUAAUUUAUCGUGAG--CUAGCUAUAGAUAUUAUGCUCAUUACUUUGGAUAUGUUGUGGAA--------------------CUUACAUC

P. aegeria(prediction) -----------AUUACAAUCCUCAACAUUUUCCAAAGUAGUGGGCGUAAUUUAUCGUGAG--CUAGCUAUAGAUAUUAUGCUCAUUACUUUGGAUAUGUUGUGGAA----------------------------

D. plexippus ------------------------------UCCAAAGUAGUGGACGUAAUUUAUCGUGAG--CCAACUAUAGAUAUUAUGCUCAUUACUUUGGAUAUGUUGUGCG-----------------------------

H. melpomene ---CGUAUAAUUAAUUAAUCUACAACGUCACCCAAAGUAGUGAACGUAAUUUAUCGUGAG--CUAUCUAUAGAUAUUAUGCUCAUUACUUUGGAGAAGUUGUACGAACAUUCAUCGAUGGAGAAUUUUUAUUCG

B. mori --------------------GGCAAUGUACUGCAAAGUAGUGAACAUAAUUUAUCGUGAGAUUUAUUAUUAGAUAUUAUGCUCAUUUCUUUGGAUAUGUUGUACG-----------------------------

M. sexta --------UACAAUACACUCGGCAACUUACUCCAAAGUAGUGAACGUAAUUUAUCGUGAGAUCAAUCUAUAGAUAUUAUGCUCAUUACUUUGGAUAUGUUGUGCGAA---------------------------

P. c-album -----------AAUUUAAUCGGCUACAUUCCCCAAAGUAGUGAACGUAAUUUAUCGUGGA--AUGAGCCU-GAUAUUAUGCUCAUUACUUUGGAUAUGUUGAACGA----------------------------
