# Supplementary material for: A Diversity of Conserved and Novel Ovarian MicroRNAs in the Speckled Wood (Pararge aegeria)
Source: PLoS One. 2015 Nov 10;10(11):e0142243. doi: 10.1371/journal.pone.0142243 (PMC4640560; doi:10.1371/journal.pone.0142243)
Supplement: S2 Table — (DOCX) [file pone.0142243.s003.docx]

|  | **Pooled** | | **St Hubert (Belgium)** | | | **Zonza (Corsica)** | | |
| --- | --- | --- | --- | --- | --- | --- | --- | --- |
|  | **Total** | **Norm.** | **Total** | **Norm.** | **FC** | **Total** | **Norm.** | **FC** |
| *B-0042* | *0* | *0* | *472* | *1.26E-03* | *N.A.* | *0* | *0.00E+00* | *N.A.* |
| *B-0070/C-0072* | *0* | *0* | *291* | *7.77E-04* | *N.A.* | *574* | *5.47E-04* | *N.A.* |
| *B-0112* | *0* | *0* | *173* | *4.62E-04* | *N.A.* | *0* | *0.00E+00* | *N.A.* |
| *B-0164* | *0* | *0* | *90* | *2.40E-04* | *N.A.* | *0* | *0.00E+00* | *N.A.* |
| *B-0210/C-0160* | *0* | *0* | *97* | *2.59E-04* | *N.A.* | *262* | *2.50E-04* | *N.A.* |
| *B-0218* | *0* | *0* | *80* | *2.14E-04* | *N.A.* | *0* | *0.00E+00* | *N.A.* |
| *B-0298* | *0* | *0* | *91* | *2.43E-04* | *N.A.* | *0* | *0.00E+00* | *N.A.* |
| *B-0319* | *0* | *0* | *48* | *1.28E-04* | *N.A.* | *0* | *0.00E+00* | *N.A.* |
| B-0401/C-0173 | 0 | 0 | 27 | 7.21E-05 | N.A. | 137 | 1.30E-04 | N.A. |
| *C-0149* | *0* | *0* | *0* | *0.00E+00* | *N.A.* | *378* | *3.60E-04* | *N.A.* |
| *C-0261* | *0* | *0* | *0* | *0.00E+00* | *N.A.* | *56* | *5.33E-05* | *N.A.* |
| Pae-bantam | 6734 | 1.86E-03 | 576 | 1.54E-03 | 0.8 | 771 | 7.34E-04 | 0.4 |
| Pae-let-7 | 471778 | 1.30E-01 | 1895 | 5.06E-03 | 0.0 | 6133 | 5.84E-03 | 0.0 |
| Pae-miR-1 | 1611661 | 4.45E-01 | 21127 | 5.64E-02 | 0.1 | 26920 | 2.56E-02 | 0.1 |
| Pae-miR-1000 | 125 | 3.45E-05 | 0 | 0.00E+00 | 0.0 | 0 | 0.00E+00 | 0.0 |
| Pae-miR-10a | 78187 | 2.16E-02 | 473 | 1.26E-03 | 0.1 | 658 | 6.27E-04 | 0.0 |
| Pae-miR-11 | 1412 | 3.90E-04 | 79 | 2.11E-04 | 0.5 | 227 | 2.16E-04 | 0.6 |
| Pae-miR-1175 | 310 | 8.56E-05 | 0 | 0.00E+00 | 0.0 | 0 | 0.00E+00 | 0.0 |
| Pae-miR-12 | 849 | 2.34E-04 | 47 | 1.26E-04 | 0.5 | 103 | 9.81E-05 | 0.4 |
| Pae-miR-124 | 1265 | 3.49E-04 | 4 | 1.07E-05 | 0.0 | 23 | 2.19E-05 | 0.1 |
| Pae-miR-133 | 279 | 7.70E-05 | 9 | 2.40E-05 | 0.3 | 0 | 0.00E+00 | 0.0 |
| Pae-miR-137 | 23 | 6.35E-06 | 0 | 0.00E+00 | 0.0 | 0 | 0.00E+00 | 0.0 |
| Pae-miR-13a | 1534 | 4.23E-04 | 38 | 1.01E-04 | 0.2 | 88 | 8.38E-05 | 0.2 |
| Pae-miR-13b | 333 | 9.19E-05 | 15 | 4.01E-05 | 0.4 | 42 | 4.00E-05 | 0.4 |
| Pae-miR-184 | 136896 | 3.78E-02 | 4805 | 1.28E-02 | 0.3 | 10233 | 9.75E-03 | 0.3 |
| Pae-miR-190 | 4251 | 1.17E-03 | 585 | 1.56E-03 | 1.3 | 800 | 7.62E-04 | 0.6 |
| Pae-miR-193 | 183 | 5.05E-05 | 0 | 0.00E+00 | 0.0 | 0 | 0.00E+00 | 0.0 |
| Pae-miR-210 | 513 | 1.42E-04 | 0 | 0.00E+00 | 0.0 | 0 | 0.00E+00 | 0.0 |
| Pae-miR-252 | 6140 | 1.69E-03 | 19 | 5.07E-05 | 0.0 | 115 | 1.10E-04 | 0.1 |
| Pae-miR-263a | 53155 | 1.47E-02 | 24821 | 6.63E-02 | 4.5 | 63593 | 6.06E-02 | 4.1 |
| Pae-miR-263b | 54 | 1.49E-05 | 0 | 0.00E+00 | 0.0 | 0 | 0.00E+00 | 0.0 |
| Pae-miR-274 | 64 | 1.77E-05 | 0 | 0.00E+00 | 0.0 | 0 | 0.00E+00 | 0.0 |
| Pae-miR-2755 | 56422 | 1.56E-02 | 4440 | 1.19E-02 | 0.8 | 10312 | 9.82E-03 | 0.6 |
| Pae-miR-2756 | 26489 | 7.31E-03 | 1406 | 3.76E-03 | 0.5 | 3516 | 3.35E-03 | 0.5 |
| Pae-miR-276 | 36461 | 1.01E-02 | 384 | 1.03E-03 | 0.1 | 580 | 5.52E-04 | 0.1 |
| Pae-miR-2765 | 4745 | 1.31E-03 | 1 | 2.67E-06 | 0.0 | 42 | 4.00E-05 | 0.0 |
| Pae-miR-2766 | 88117 | 2.43E-02 | 1628 | 4.35E-03 | 0.2 | 4035 | 3.84E-03 | 0.2 |
| Pae-miR-2767 | 39977 | 1.10E-02 | 51 | 1.36E-04 | 0.0 | 1224 | 1.17E-03 | 0.1 |
| Pae-miR-2768 | 452 | 1.25E-04 | 0 | 0.00E+00 | 0.0 | 0 | 0.00E+00 | 0.0 |
| Pae-miR-277 | 4616 | 1.27E-03 | 81 | 2.16E-04 | 0.2 | 106 | 1.01E-04 | 0.1 |
| Pae-miR-278 | 9065 | 2.50E-03 | 625 | 1.67E-03 | 0.7 | 1536 | 1.46E-03 | 0.6 |
| Pae-miR-2788 | 105 | 2.90E-05 | 0 | 0.00E+00 | 0.0 | 0 | 0.00E+00 | 0.0 |
| Pae-miR-2796 | 5006 | 1.38E-03 | 2 | 5.34E-06 | 0.0 | 21 | 2.00E-05 | 0.0 |
| Pae-miR-2797a | 9503 | 2.62E-03 | 0 | 0.00E+00 | 0.0 | 0 | 0.00E+00 | 0.0 |
| Pae-miR-2797b | 6120 | 1.69E-03 | 0 | 0.00E+00 | 0.0 | 0 | 0.00E+00 | 0.0 |
| Pae-miR-2797c | 2002 | 5.53E-04 | 0 | 0.00E+00 | 0.0 | 0 | 0.00E+00 | 0.0 |
| Pae-miR-279a | 18068 | 4.99E-03 | 1854 | 4.95E-03 | 1.0 | 3188 | 3.04E-03 | 0.6 |
| Pae-miR-279b | 30680 | 8.47E-03 | 2051 | 5.48E-03 | 0.6 | 4564 | 4.35E-03 | 0.5 |
| Pae-miR-279c | 847 | 2.34E-04 | 53 | 1.42E-04 | 0.6 | 124 | 1.18E-04 | 0.5 |
| Pae-miR-279d | 27122 | 7.49E-03 | 2374 | 6.34E-03 | 0.8 | 4410 | 4.20E-03 | 0.6 |
| Pae-miR-281 | 47901 | 1.32E-02 | 1292 | 3.45E-03 | 0.3 | 2067 | 1.97E-03 | 0.1 |
| Pae-miR-282 | 852 | 2.35E-04 | 0 | 0.00E+00 | 0.0 | 0 | 0.00E+00 | 0.0 |
| Pae-miR-283 | 156 | 4.31E-05 | 0 | 0.00E+00 | 0.0 | 0 | 0.00E+00 | 0.0 |
| Pae-miR-285 | 2503 | 6.91E-04 | 3 | 8.01E-06 | 0.0 | 7 | 6.67E-06 | 0.0 |
| Pae-miR-2a | 9979 | 2.75E-03 | 111 | 2.96E-04 | 0.1 | 424 | 4.04E-04 | 0.1 |
| Pae-miR-2b | 2183 | 6.02E-04 | 37 | 9.88E-05 | 0.2 | 60 | 5.71E-05 | 0.1 |
| Pae-miR-2c | 10186 | 2.81E-03 | 71 | 1.90E-04 | 0.1 | 162 | 1.54E-04 | 0.1 |
| Pae-miR-3000 | 46 | 1.27E-05 | 0 | 0.00E+00 | 0.0 | 0 | 0.00E+00 | 0.0 |
| Pae-miR-307 | 415 | 1.15E-04 | 12 | 3.20E-05 | 0.3 | 15 | 1.43E-05 | 0.1 |
| Pae-miR-308 | 5385 | 1.49E-03 | 576 | 1.54E-03 | 1.0 | 1608 | 1.53E-03 | 1.0 |
| Pae-miR-31 | 64433 | 1.78E-02 | 959 | 2.56E-03 | 0.1 | 2418 | 2.30E-03 | 0.1 |
| Pae-miR-316 | 130 | 3.59E-05 | 8 | 2.14E-05 | 0.6 | 0 | 0.00E+00 | 0.0 |
| Pae-miR-317 | 6220 | 1.72E-03 | 512 | 1.37E-03 | 0.8 | 598 | 5.70E-04 | 0.3 |
| Pae-miR-3286 | 323 | 8.91E-05 | 0 | 0.00E+00 | 0.0 | 0 | 0.00E+00 | 0.0 |
| Pae-miR-33 | 254 | 7.01E-05 | 12 | 3.20E-05 | 0.5 | 18 | 1.71E-05 | 0.2 |
| Pae-miR-3338 | 21 | 5.80E-06 | 0 | 0.00E+00 | 0.0 | 13 | 1.24E-05 | 2.1 |
| Pae-miR-34 | 13370 | 3.69E-03 | 657 | 1.75E-03 | 0.5 | 956 | 9.11E-04 | 0.2 |
| Pae-miR-375 | 1217 | 3.36E-04 | 6 | 1.60E-05 | 0.0 | 20 | 1.90E-05 | 0.1 |
| Pae-miR-6094 | 496 | 1.37E-04 | 0 | 0.00E+00 | 0.0 | 0 | 0.00E+00 | 0.0 |
| Pae-miR-6307 | 624 | 1.72E-04 | 59 | 1.58E-04 | 0.9 | 132 | 1.26E-04 | 0.7 |
| Pae-miR-7 | 391 | 1.08E-04 | 44 | 1.18E-04 | 1.1 | 43 | 4.10E-05 | 0.4 |
| Pae-miR-745 | 1883 | 5.20E-04 | 19 | 5.07E-05 | 0.1 | 40 | 3.81E-05 | 0.1 |
| Pae-miR-750 | 1945 | 5.37E-04 | 0 | 0.00E+00 | 0.0 | 0 | 0.00E+00 | 0.0 |
| Pae-miR-8 | 634 | 1.75E-04 | 78 | 2.08E-04 | 1.2 | 290 | 2.76E-04 | 1.6 |
| Pae-miR-87 | 346 | 9.55E-05 | 54 | 1.44E-04 | 1.5 | 63 | 6.00E-05 | 0.6 |
| Pae-miR-9 | 9534 | 2.63E-03 | 130 | 3.47E-04 | 0.1 | 217 | 2.07E-04 | 0.1 |
| Pae-miR-927 | 167 | 4.61E-05 | 0 | 0.00E+00 | 0.0 | 0 | 0.00E+00 | 0.0 |
| Pae-miR-929 | 11 | 3.04E-06 | 0 | 0.00E+00 | 0.0 | 0 | 0.00E+00 | 0.0 |
| Pae-miR-92a | 6220 | 1.72E-03 | 198 | 5.29E-04 | 0.3 | 426 | 4.06E-04 | 0.2 |
| Pae-miR-92b | 1640 | 4.53E-04 | 0 | 0.00E+00 | 0.0 | 110 | 1.05E-04 | 0.2 |
| Pae-miR-932 | 126 | 3.48E-05 | 0 | 0.00E+00 | 0.0 | 0 | 0.00E+00 | 0.0 |
| Pae-miR-965 | 352 | 9.71E-05 | 32 | 8.55E-05 | 0.9 | 52 | 4.95E-05 | 0.5 |
| Pae-miR-970 | 8319 | 2.30E-03 | 194 | 5.18E-04 | 0.2 | 593 | 5.65E-04 | 0.2 |
| Pae-miR-971 | 18 | 4.97E-06 | 0 | 0.00E+00 | 0.0 | 0 | 0.00E+00 | 0.0 |
| Pae-miR-981 | 18 | 4.97E-06 | 0 | 0.00E+00 | 0.0 | 0 | 0.00E+00 | 0.0 |
| Pae-miR-988 | 84 | 2.32E-05 | 9 | 2.40E-05 | 1.0 | 13 | 1.24E-05 | 0.5 |
| Pae-miR-989 | 538441 | 1.49E-01 | 288099 | 7.69E-01 | 5.2 | 873884 | 8.32E-01 | 5.6 |
| Pae-miR-993 | 37297 | 1.03E-02 | 4611 | 1.23E-02 | 1.2 | 8301 | 7.91E-03 | 0.8 |
| Pae-miR-998 | 1389 | 3.83E-04 | 52 | 1.39E-04 | 0.4 | 127 | 1.21E-04 | 0.3 |
| Pae-miR-iab-4 | 706 | 1.95E-04 | 15 | 4.01E-05 | 0.2 | 33 | 3.14E-05 | 0.2 |
| Pae-miR-iab-8 | 30 | 8.28E-06 | 0 | 0.00E+00 | 0.0 | 0 | 0.00E+00 | 0.0 |
| *Par-013* | *42783* | *1.18E-02* | *1714* | *4.58E-03* | *0.4* | *4723* | *4.50E-03* | *0.4* |
| *Par-017* | *41987* | *1.16E-02* | *1935* | *5.17E-03* | *0.4* | *5586* | *5.32E-03* | *0.5* |
| *Par-058* | *2394* | *6.61E-04* | *185* | *4.94E-04* | *0.7* | *342* | *3.26E-04* | *0.5* |
| *Par-080* | *810* | *2.24E-04* | *70* | *1.87E-04* | *0.8* | *171* | *1.63E-04* | *0.7* |
| *Par-094* | *100* | *2.76E-05* | *0* | *0.00E+00* | *0.0* | *128* | *1.22E-04* | *4.4* |
| *Par-099* | *640* | *1.77E-04* | *46* | *1.23E-04* | *0.7* | *91* | *8.67E-05* | *0.5* |
| *Par-104* | *905* | *2.50E-04* | *38* | *1.01E-04* | *0.4* | *73* | *6.95E-05* | *0.3* |
| Par-110 | 663 | 1.83E-04 | 0 | 0.00E+00 | 0.0 | 0 | 0.00E+00 | 0.0 |
| *Par-111* | *291* | *8.03E-05* | *85* | *2.27E-04* | *2.8* | *128* | *1.22E-04* | *1.5* |
| *Par-117* | *673* | *1.86E-04* | *0* | *0.00E+00* | *0.0* | *0* | *0.00E+00* | *0.0* |
| *Par-118* | *82* | *2.26E-05* | *19* | *5.07E-05* | *2.2* | *67* | *6.38E-05* | *2.8* |
| *Par-129* | *752* | *2.08E-04* | *148* | *3.95E-04* | *1.9* | *577* | *5.50E-04* | *2.6* |
| *Par-145* | *373* | *1.03E-04* | *237* | *6.33E-04* | *6.1* | *0* | *0.00E+00* | *0.0* |
| *Par-146* | *333* | *9.19E-05* | *11* | *2.94E-05* | *0.3* | *60* | *5.71E-05* | *0.6* |
| *Par-152* | *372* | *1.03E-04* | *0* | *0.00E+00* | *0.0* | *0* | *0.00E+00* | *0.0* |
| *Par-160* | *248* | *6.84E-05* | *0* | *0.00E+00* | *0.0* | *0* | *0.00E+00* | *0.0* |
| *Par-168* | *365* | *1.01E-04* | *0* | *0.00E+00* | *0.0* | *60* | *5.71E-05* | *0.6* |
| *Par-181* | *144* | *3.97E-05* | *0* | *0.00E+00* | *0.0* | *8* | *7.62E-06* | *0.2* |
| *Par-189* | *87* | *2.40E-05* | *0* | *0.00E+00* | *0.0* | *0* | *0.00E+00* | *0.0* |
| *Par-211* | *114* | *3.15E-05* | *11* | *2.94E-05* | *0.9* | *0* | *0.00E+00* | *0.0* |
| *Par-212* | *125* | *3.45E-05* | *63* | *1.68E-04* | *4.9* | *0* | *0.00E+00* | *0.0* |
| *Par-220* | *95* | *2.62E-05* | *3* | *8.01E-06* | *0.3* | *10* | *9.52E-06* | *0.4* |
| *Par-222* | *81* | *2.24E-05* | *57* | *1.52E-04* | *6.8* | *14* | *1.33E-05* | *0.6* |
| *Par-240* | *75* | *2.07E-05* | *21* | *5.61E-05* | *2.7* |  | *0.00E+00* | *0.0* |
| *Par-247* | *44* | *1.21E-05* | *125* | *3.34E-04* | *27.5* | *146* | *1.39E-04* | *11.5* |
| *Par-259* | *77* | *2.13E-05* | *0* | *0.00E+00* | *0.0* | *0* | *0.00E+00* | *0.0* |
| *Par-262* | *59* | *1.63E-05* | *0* | *0.00E+00* | *0.0* | *0* | *0.00E+00* | *0.0* |
| *Par-268* | *47* | *1.30E-05* | *0* | *0.00E+00* | *0.0* | *0* | *0.00E+00* | *0.0* |
| *Par-269* | *92* | *2.54E-05* | *0* | *0.00E+00* | *0.0* | *0* | *0.00E+00* | *0.0* |
| *Par-280* | *57* | *1.57E-05* | *0* | *0.00E+00* | *0.0* | *0* | *0.00E+00* | *0.0* |
| *Par-284* | *54* | *1.49E-05* | *15* | *4.01E-05* | *2.7* | *29* | *2.76E-05* | *1.9* |
| *Par-290* | *33* | *9.11E-06* | *8* | *2.14E-05* | *2.3* | *0* | *0.00E+00* | *0.0* |
| *Par-305* | *51* | *1.41E-05* | *6* | *1.60E-05* | *1.1* | *0* | *0.00E+00* | *0.0* |
| *Par-324* | *50* | *1.38E-05* | *0* | *0.00E+00* | *0.0* | *9* | *8.57E-06* | *0.6* |
| *Par-340* | *49* | *1.35E-05* | *52* | *1.39E-04* | *10.3* | *70* | *6.67E-05* | *4.9* |
| *Par-341* | *48* | *1.32E-05* | *52* | *1.39E-04* | *10.5* | *70* | *6.67E-05* | *5.0* |
| *Par-350* | *21* | *5.80E-06* | *0* | *0.00E+00* | *0.0* | *0* | *0.00E+00* | *0.0* |
| *Par-371* | *23* | *6.35E-06* | *0* | *0.00E+00* | *0.0* | *0* | *0.00E+00* | *0.0* |
| *Par-377* | *19* | *5.24E-06* | *3* | *8.01E-06* | *1.5* | *0* | *0.00E+00* | *0.0* |
| *Par-404* | *19* | *5.24E-06* | *0* | *0.00E+00* | *0.0* | *0* | *0.00E+00* | *0.0* |
| *Par-405* | *19* | *5.24E-06* | *0* | *0.00E+00* | *0.0* | *0* | *0.00E+00* | *0.0* |
| *Par-425* | *38* | *1.05E-05* | *0* | *0.00E+00* | *0.0* | *0* | *0.00E+00* | *0.0* |
| *Par-426* | *36* | *9.94E-06* | *13* | *3.47E-05* | *3.5* | *0* | *0.00E+00* | *0.0* |
| *Par-471* | *27* | *7.45E-06* | *0* | *0.00E+00* | *0.0* | *0* | *0.00E+00* | *0.0* |
| *Par-523* | *20* | *5.52E-06* | *0* | *0.00E+00* | *0.0* | *0* | *0.00E+00* | *0.0* |
| *Par-536* | *10* | *2.76E-06* | *0* | *0.00E+00* | *0.0* | *0* | *0.00E+00* | *0.0* |
| *Par-542* | *11* | *3.04E-06* | *0* | *0.00E+00* | *0.0* | *97* | *9.24E-05* | *30.4* |
| *Par-543* | *14* | *3.86E-06* | *0* | *0.00E+00* | *0.0* | *5* | *4.76E-06* | *1.2* |
| *Par-554* | *14* | *3.86E-06* | *0* | *0.00E+00* | *0.0* | *0* | *0.00E+00* | *0.0* |
| *Par-567* | *15* | *4.14E-06* | *0* | *0.00E+00* | *0.0* | *0* | *0.00E+00* | *0.0* |
| *Par-602* | *11* | *3.04E-06* | *0* | *0.00E+00* | *0.0* | *0* | *0.00E+00* | *0.0* |
| *Par-605* | *7* | *1.93E-06* | *0* | *0.00E+00* | *0.0* | *0* | *0.00E+00* | *0.0* |
| *Par-614* | *8* | *2.21E-06* | *0* | *0.00E+00* | *0.0* | *0* | *0.00E+00* | *0.0* |
| *Par-634* | *17* | *4.69E-06* | *0* | *0.00E+00* | *0.0* | *0* | *0.00E+00* | *0.0* |
| *Par-658* | *6* | *1.66E-06* | *0* | *0.00E+00* | *0.0* | *0* | *0.00E+00* | *0.0* |
| *Par-659* | *16* | *4.42E-06* | *6* | *1.60E-05* | *3.6* | *0* | *0.00E+00* | *0.0* |
| *Par-700* | *10* | *2.76E-06* | *0* | *0.00E+00* | *0.0* | *0* | *0.00E+00* | *0.0* |
| *Par-737* | *3* | *8.28E-07* | *0* | *0.00E+00* | *0.0* | *0* | *0.00E+00* | *0.0* |
| *Par-738* | *9* | *2.48E-06* | *5* | *1.34E-05* | *5.4* | *0* | *0.00E+00* | *0.0* |

Read count comparison data for the pooled miRNA transcriptome (Quah et al., 2015) and the ovarian transcriptomes from St Hubert and Zonza. Read counts are normalised to the total number of reads mapped as miRNAs in each transcriptome. FC = fold change. Italics indicate lineage-specific miRNAs.
